# Perioperative Evaluation of Patient Outcomes after Severe Acid Corrosive Injury

**DOI:** 10.1155/2015/545262

**Published:** 2015-10-25

**Authors:** Ming-Ho Wu, Han-Yun Wu

**Affiliations:** Department of Surgery, Tainan Municipal Hospital, 670 Chung-Te Road, Tainan 701, Taiwan

## Abstract

We reviewed 64 patients with perforation or full-thickness injury of the alimentary tract after acid ingestion. Based on our classification of laparotomy findings, there were class I (*n* = 15); class II (*n* = 13); class III (*n* = 16); and class IV (*n* = 20). Study parameters were preoperative laboratory data, gastric perforation, associated visceral injury, and extension of the injury. End points of the study were the patients' mortality and length of hospital stay. All these patients underwent esophagogastrectomy with (*n* = 16) or without (*n* = 24) concomitant resection, esophagogastroduodenojejunectomy with (*n* = 4) or without (*n* = 13) concomitant resection, and laparotomy only (*n* = 7). Concomitant resections were performed on the spleen (*n* = 10), colon (*n* = 2), pancreas (*n* = 1), gall bladder (*n* = 1), skipped areas of jejunum (*n* = 4), and the first portion of the duodenum (*n* = 4). The study demonstrates five preoperative risk factors, female gender, shock status, shock index, pH value, and base deficit, and four intraoperative risk factors, gastric perforation, associated visceral injury, injury beyond the pylorus, and continuous involvement of the jejunum over a length of 50 cm. The overall mortality rate was 45.3%, which increased significantly with advancing class of corrosive injury.

## 1. Introduction

Corrosive injury could be accidental or suicide attempt. Ingested agents include acid or alkali [1]. Acid ingestion is one of the most frequent means of attempted suicide in Taiwan. Ingestion of strong acids often results in a corrosive injury to the upper alimentary tract [2, 3]. Isolated gastric outlet obstruction will be detected several weeks later in some moderately injured patients [4]. In some severely injured patients, associated injuries of intra-abdominal organs, such as the pancreas, gall bladder, spleen, colon, diaphragm, and skipped areas of jejunum, occur frequently. Aggressive surgery may save the life of extensively injured patients during the acute stage [5–13]. However, the mortality rate varies in surgically treated patients, ranging from 40% to 77.7% [7, 10, 11, 14]. It is clinically important to identify intraoperative risk factors and to form subgroups according to risk of death so that injuries can be categorized. In the study, the preoperative clinical data, intraoperative risk factors, surgical procedures, outcomes, and follow-up data of patients with extensive corrosive injuries were reviewed retrospectively from medical charts. The obtained data were analyzed to categorize laparotomy findings to evaluate the patient outcomes.

## 2. Materials and Methods

### 2.1. Management of Acute Corrosive Injury

During the acute stage of extensive corrosive injury, treatment includes fasting, nasogastric tube decompression, intravenous fluid replacement, and correction of any acid-base imbalance and antibiotics and H_2_-blockers. Complete blood cell counts, leukocyte differential counts, blood biochemistry analysis, and chest and plain abdominal film studies are routinely conducted. Arterial blood gases are regularly monitored. Endoscopy is performed only on patients who do not require immediate surgery. Comatose or hypoxic patients are intubated immediately for airway security. Ventilatory support is provided for patients with respiratory failure.

### 2.2. Selection of Patients for Laparotomy

An early exploratory laparotomy is mandatory in the presence of generalized peritoneal signs, continuous gastrointestinal bleeding, endoscopic findings of severe burns of the esophagus and stomach, pleural effusion, hydropneumothorax, or pneumoperitoneum determined by radiographic examination, or pH < 7.0 [5] or a base deficit > 16 mmol/L on initial arterial blood gases (ABG) analysis.

### 2.3. Definition of Severe Corrosive Injury

In this study, severe corrosive injury is defined as perforation or full-thickness injury of the alimentary tract.

### 2.4. Classification of Severe Acid Corrosive Injury Based on Laparotomy Findings (Table 1)

The associated visceral injury represents the tissue damage of the pancreas, gall bladder, spleen, colon, diaphragm, liver, or skipped areas of jejunum. In cases of previous subtotal gastrectomy associated with gastrojejunostomy, the jejunum connected to the stomach was considered as a continuous involvement when it was injured. An estimate of the injury in patients who had had a previous gastrectomy appeared to have been defined a little differently. In gastrectomy patients, continuous involvement of the jejunum longer than 30 cm was comparable to injury beyond the pylorus in normal individuals, that longer than 50 cm was comparable to injury beyond the duodenum, and that longer than 100 cm was comparable to beyond 50 cm of the jejunum in the normal individual.

### 2.5. Methods

Medical records were reviewed retrospectively on preoperative (Table 2) and intraoperative (Table 3) risk factors. The parameters were age (<45 versus ≥45 years), gender, shock (BP < 90 mmHg), shock index (<1 versus ≥1), white blood cell count (<15000 versus ≥15000/*μ*L), hemoglobin (<13.5 versus ≥13.5 g/dL), pH (<7.23 versus ≥7.23), base deficit (<14 versus ≥14 mmol/L), amylase (<130 versus ≥130 IU/L), gastric perforation, associated visceral injury, injury beyond the pylorus, and continuous involvement of the jejunum over a length of 50 cm. The selection of the cut-off values for white blood cell count, hemoglobin, pH, base deficit, amylase, and length of the jejunum was based on a median of the respective data. Analysis was performed mainly to identify the above four intraoperative risk factors. The patients in the series were categorized by using the laparotomy findings according to the rule described above. End points of the study were the patients' mortality and their length of hospital stay.

### 2.6. Statistical Analysis

Continuous variables are expressed as the mean ± the standard error. The Chi-square test was used to determine the significance of the difference between categorical variables, including the preoperative and intraoperative variables, surgical procedures, outcomes, and length of hospital stay. A value of *p* < 0.05 was considered as statistically significant. These analyses were performed using the SPSS 12.0 software package for Windows (SPSS, Inc., Chicago, IL).

## 3. Results

### 3.1. Patients Who Underwent Emergency Surgery for Extensive Corrosive Injury

A total of 426 patients with acid corrosive injuries were treated in a period of 14 years. Of those, 84 (19.7%) underwent surgery in the acute stage according to the selection criteria described above. Twenty (4.6%) patients who underwent gastrostomy and jejunostomy only, not recognized as a severe injury, were excluded from the study. The remaining 64 (15%) patients (28 men and 36 women) ranged from 16 to 78 years old (mean + SEM, 46.3 ± 1.9 years old). All these patients had ingested acid in a liquid form, mostly hydrochloric acid. The volume of acid ingested varied from 50 to 450 mL in 10~30% concentrations. Sixty-one (95.3%) of the patients ingested the caustics in suicidal attempts. The interval between the injury and laparotomy was 14.6 ± 2.6 hours.

### 3.2. Operative Procedure

**Figure 1:**
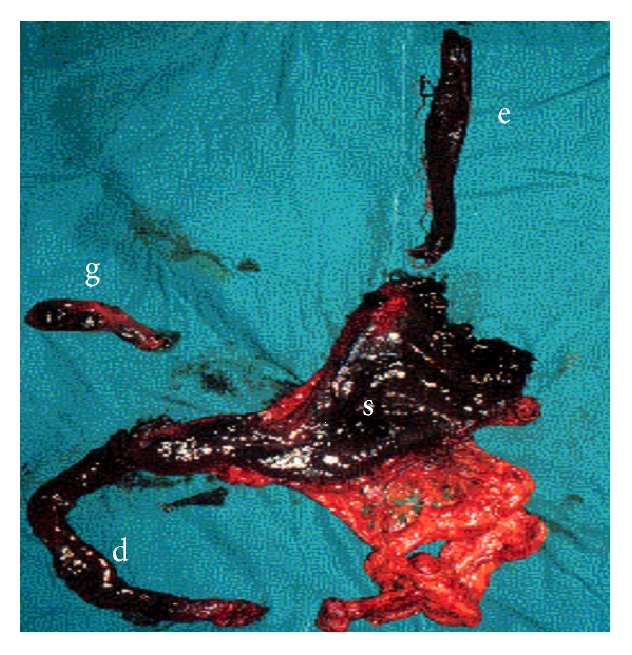
These organs were resected from a 32-year-old woman with severe acid corrosive injury; e: esophagus, s: perforated stomach, g: gall bladder, and d: duodenum.

The surgical procedures consisted of esophagogastrectomy with or without concomitant resection, esophagogastroduodenojejunectomy with or without concomitant resection (Figure 1), and exploratory laparotomy only. Concomitant resection of the associated viscera was performed as indications stipulated. Cervical esophagostomy and feeding jejunostomy were also performed in cases of resection. All resections were performed via midline laparotomy and oblique neck incision. The esophagectomy was initially performed via the transhiatal route in all patients. Because thrombosis of the periesophageal vessels always occurred and there was no obvious esophageal perforation following acid ingestion in this series, only one patient needed a thoracotomy as a result of massive intrathoracic bleeding.

### 3.3. Postoperative Care and Follow-Up

All patients received postoperative care in an intensive care unit either until they were weaned from ventilators and their vital signs were stable or until death. Once patients had had a bowel movement or a flatus passage, feeding via jejunostomy was started. Total parenteral nutrition was reserved for patients who had a paralytic ileus or intestinal complications. We continued regular outpatient follow-up after discharge and subsequently performed esophageal reconstruction for survivors.

The study demonstrated five preoperative risk factors, namely, female gender, shock status, shock index ≥ 1, pH value < 7.23, and base deficit > 14 mmol/L, and four intraoperative risk factors, namely, gastric perforation, associated visceral injury, injury beyond the pylorus, and continuous involvement of the jejunum over a length of 50 cm. Among these 64 patients, 15 (23.4%) were class I, 13 (20.3%) were class II, 16 (25.0%) were class III, and 20 (31.3%) were class IV (Table 4). They all underwent esophagogastrectomy with (*n* = 16, 25.0%) or without (*n* = 24, 37.5%) concomitant resection, esophagogastroduodenojejunectomy with (*n* = 4, 6.3%) or without (*n* = 13, 20.3%) concomitant resection, and laparotomy only (*n* = 7, 10.9%). Concomitant resections were performed on the spleen (*n* = 10), colon (*n* = 2), pancreas (*n* = 1), gall bladder (*n* = 1), skipped areas of jejunum (*n* = 4), and the first portion of the duodenum (*n* = 4). The intraoperative risk factors, including the gastric perforation, associated visceral injury, injury beyond the pylorus, and continuous involvement of the jejunum over a length of 50 cm, and operative procedure correlate significantly with death in each class of injury (*p* = 0.044, 0.000, 0.000, 0.000, and 0.000, resp.). The overall mortality rate was 45.3% (29/64), which increased significantly with advancing class of corrosive injury (class I, 0.0%; class II, 23.1%; class III, 56.3%; and class IV, 85.0%, *p* = 0.000). These 29 patients died between days 1 and 64 (average 14.4 days), including 12 (41.3%) who died within 24 hours after surgery. Four deaths received cardiopulmonary resuscitation during surgery. The major causes of death were multiple organ failure (*n* = 20) and sepsis (*n* = 9). The average hospital stay of 35 (54.7%) survivors was 44.8 ± 7.1 days, with a significant increase with advancing class of injury (*p* = 0.000). The range of follow-up for survivors was from 6 months to 9 years, with a median of 3.9 years. Twenty-nine (45.3%) patients subsequently underwent reconstruction of the esophagus, from 2.5 months to 8 months (average of 3.5 months) after an esophagogastrectomy (*n* = 25) or an esophagogastroduodenojejunectomy (*n* = 4).

## 4. Discussion

All of the series of patients had extensive corrosive injuries after ingesting strong acids. Their presentations, management, and injury patterns were different from those of patients with alkali injury, which commonly occurs in western countries. Previous reports [14, 15] revealed that most deaths from alkali injury are due to associated intrathoracic organ injuries, such as tracheobronchial necrosis or esophagoaortic fistula. In our series, females had a higher risk of death. Probably, females ingested larger amounts of the corrosive agent (255.6 ± 18.6 mL versus 229.7 ± 16.5 mL, *p* = 0.356). A shock index ≥ 1 and coma in the emergency room were two ominous signs [7, 10, 15] for a poor prognosis because they were an indication of associated intra-abdominal organ injuries. A shock index ≥ 1 indicated severe fluid loss from the third space of the abdominal cavity. Most of these patients were in poor general condition as a result of extensive associated organ injuries. Patients with irreversible coma usually die quite soon. Patients presenting with a shock index ≥ 1 in the emergency room should undergo aggressive perioperative fluid resuscitation and acid-base correction, followed by adequate resection of injured organs as soon as possible, although the mortality rate is extremely high.

In acid-injured patients, a blood pH value < 7.0 usually indicates the necessity for esophagogastrectomy [7]. In this study, a blood pH value < 7.23 was significantly related to deaths compared to a pH value ≥ 7.23. A base deficit* in vivo* or within the extracellular fluid has long been used as the index of a nonrespiratory acid-base imbalance [16]. In our animal experiment [17], obvious gastric perforation, which caused intra-abdominal organ injuries, occurred in animals that had a base deficit of more than 16 mmol/L. In the present study, a base deficit of more than 14 mmol/L is a significant predictor of death (*p* = 0.009). We highly recommend that patients with a base deficit of more than 14 mmol/L on initial ABG also undergo early surgery. Endoscopy has been used for routine evaluation of corrosive injury [18]. In our institute, the endoscopy is performed only on patients who do not require immediate surgery.

The present study showed that four intraoperative findings were well correlated to the deaths in each class of acid corrosive injury. These findings were (1) gastric perforation which reflects severe injury of the gastric wall; (2) associated visceral injury which reflects extra-alimentary extension of injury; (3) injury beyond the pylorus, and (4) continuous involvement of the jejunum over a length of 50 cm, the last two findings reflecting intra-alimentary extension of the injury. Any combination of the above findings always made the treatment more complicated and the patient outcome worse. According to the analysis of the intraoperative risk factors and the surgeon's experience, categorization of severe acid corrosive injury should be mandatory.

In the series, 56.5% (26 of 46) of gastric perforations had associated visceral injuries, compared to 38.8% (7/18) of nongastric perforations which had associated visceral injuries (odds ratio for associated injury = 2.043, *p* = 0.204). Obviously, the associated injuries were caused either by outflow or by direct infiltration of caustic agents.

In our experience, upper and lower abdominal pain were the most common presentation, while peritoneal signs or muscle guarding was detected less often. To improve the survival rate, we emphasize that patients with an obvious abdominal presentation should undergo early exploratory laparotomy. Left pleural effusion indicates gastric fundus perforation and also helps in the decision for surgery. In some patients, severe acid injury could have been blocked by prepyloric spasm [19] causing early perforation of the stomach, especially on the posterior wall of the fundus and body. The associated injury of the pancreas and retroperitoneal tissues precluded adequate resection. These patients often had persistent metabolic acidosis or early acute renal failure postoperatively and subsequently died from multiple organ failure. Some developed repeated internal bleeding from necrosis of the retroperitoneal tissues. Diffuse oozing in the abdominal cavity made surgical homeostasis unsuccessful. One should preserve the omentum as far as possible to cover these injured tissues to attenuate this complication. Although associated visceral injury was a major risk factor for death, some patients could be saved if the injured organs could be resected completely [20].

The esophagectomy was initially performed via the transhiatal route in all patients to lessen the morbidity and mortality [21–23]. Thoracotomy for esophagectomy was reserved for those patients who had esophageal perforation or had complications such as esophageal disruption or massive bleeding during transhiatal esophageal stripping. In the case of previous subtotal gastrectomy associated with gastrojejunostomy when the jejunum was injured, which is recognized as continuous involvement, the injury should not be ignored. In the series, three patients, with a history of subtotal gastrectomy associated with gastrojejunostomy, had continued jejunum involvement of 20 cm, 29 cm, and 120 cm, respectively. Combined diaphragm injury seemed to delay the morbidity of the survivors and some of them required repeat surgery for subphrenic, pleural, and gastro- or colobronchial fistula complications. Wide and adequate resection of injured organs is still the only way to block the process of acid injury and to improve the survival rate [8, 12, 20].

Injuries that required duodenectomy actually had a significantly poor prognosis compared to those without duodenectomy (*p* = 0.000). Most deaths were due to respiratory or intra-abdominal complications that resulted from a technically difficult surgery and complicated postoperative course [8, 9, 12]. Adequate debridement of an injured pancreas head after duodenectomy with multiple intra-abdominal and intraluminal drainage (pancreatic duct and jejunum) was performed in this series. Although pancreaticojejunostomy leakage was still found in more than one-third of the patients, 41.1% (7/17) of our patients who underwent esophagogastroduodenojejunectomy survived. However, the patients who required continuous resection of the alimentary tract beyond 50 cm of the jejunum usually had a much higher mortality, namely, 93.8% (15/16), compared to those with less than 50 cm of the jejunum involved, namely, 33.3% (3/9), *p* = 0.001.

In the early deaths, the patients had multiple visceral damage, thrombosis of the visceral vessels resulting from spreading out of the ingested agent, or extensive alimentary tract injury. Surgical procedures were halted owing to persistent unstable vital signs or disseminated bleeding. For most of those patients devitalization of all abdominal viscera made radical resection impossible [6, 7, 22]. These patients manifested diffuse abdominal pain, either with early peritoneal signs or without rebounding as seen in ischemic bowel disease, and usually died shortly after surgery.

A poor patient outcome is significantly increased in the advanced class of corrosive injury, which reflects that the risk of death is increased in severely injured patients. In the study, 29 (45.3%) patients died between days 1 and 64 (average 14.4 days), including 12 (41.3%) who died within 24 hours after surgery. Hence, only 35 (54.7%) survivors were enrolled to analyze the hospital stay, which increased significantly with advancing class of injury (*p* = 0.000).

## 5. Conclusions

Surgery remains the only way to save the life of patients with extensive corrosive injuries. Gastric perforation, associated visceral injury, and extension of the alimentary injury are well correlated with the mortality rate among each class of injury. The severity classification of acid corrosive injuries based on laparotomy findings firstly designed by us is useful to predict the probability of survival when a laparotomy is performed.

## Figures and Tables

**Table 1:** Classification of severe acid corrosive injury.

Class I	Isolated gastric full-thickness injury

Class II	(1) Gastric perforation or (2) Full-thickness injury extending from the stomach to the duodenum

Class III	(1) Injury extending from the stomach to the duodenum with perforation or (2) Full-thickness injury extending from the stomach up to 50 cm of the jejunum

Class IV	(1) Injury extending from the stomach beyond the duodenum with perforation or (2) Injury extending from the stomach beyond 50 cm of the jejunum

**Table 2:** Analysis of preoperative risk factors in 64 severely injured patients.

	Survivor	Death	*p *value
Age (years)			NS
<45	21	12	
≥45	14	17	
Gender			0.016
Male	20	8	
Female	15	21	
Shock (BP < 90 mmHg)			0.01
Yes	0	6	
No	35	23	
Shock index			0.005
≥1	3	11	
<1	32	18	
White blood cell count (/μL)			NS
<15000	14	15	
≥15000	19	13	
Platelet (k/L)^*∗*^			NS
<150	16	15	
≥150	15	12	
pH^*∗*^			0.027
<7.23	11	16	
≥7.23	21	12	
Base deficit (mmol/L)^*∗*^			0.009
<14	21	10	
≥14	9	18	
Amylase (IU/L)^*∗*^			NS
<130	11	11	
≥130	12	11	

^*∗*^Some data not available.

NS: not significant.

**Table 3:** Analysis of intraoperative risk factors in 64 severely injured patients.

	Survivor	Death	*p *value
Injury beyond pylorus			0.000
Yes	14	29	
No	21	0	
Continuous involvement of jejunum			0.000
<50 cm	6	3	
≥50 cm	1	15	
Associated visceral injury			0.000
Yes	11	22	
No	24	7	
Gastric perforation			0.001
Yes	19	27	
No	16	2	

**Table 4:** Intraoperative risk factors, operative procedures, and patient outcome in different classes of severe corrosive injury.

	Class I (*n* = 15)	Class II (*n* = 13)	Class III (*n* = 16)	Class IV (*n* = 20)	Total (*n* = 64)	*p* value
Gastric perforation	7	9	12	18	46	0.044
Associated injury	0	7	10	16	33	0.000
Injury beyond pylorus	0	6	15	20	43	0.000
Continuous involvement of jejunum						0.000
>50 cm	0	0	0	16	16	
<50 cm	0	0	5	4	11	
Operative procedure						0.000
Esophagogastrectomy	14	6	4	0	24	
Esophagogastrectomy + concomitant resection	1	7	7	1	16	
Esophagogastroduodenojejunectomy	0	0	5	8	13	
Esophagogastroduodenojejunectomy + concomitant resection	0	0	0	4	4	
Laparotomy only	0	0	0	7	7	
Mortality rate	0	3	9	17	29	0.000
Hospital stay of survivors (day)	26.1 ± 3.7	33.9 ± 6.0	56.4 ± 15.8	144.0 ± 27.2	44.8 ± 7.1	0.000
Esophageal reconstruction subsequently	13	9	6	1	29	0.000
